# ACTH-induced stress in weaned sows impairs LH receptor expression and steroidogenesis capacity in the ovary

**DOI:** 10.1186/s12958-016-0214-5

**Published:** 2016-11-14

**Authors:** H. S. Zhu, Z. Qian, H. L. Liu, E. D. Bao

**Affiliations:** 1 College of Veterinary Medicine, Nanjing Agricultural University, Weigang 1, Nanjing, 210095 China; 2 College of Animal Science and Technology, Nanjing Agricultural University, Nanjing, 210095 China

**Keywords:** ACTH, Stress, Steroidogenesis, LH receptor, Weanling sows

## Abstract

**Background:**

Stress has been proved to impair the porcine reproduction soundly. Endocrine disruption, which is closely related to the persistent follicles, is possibly one of the results of stress, although the mechanism is unclear. Since the expression of luteinizing hormone receptor (LHR) in ovarian follicular wall and concentrations of steroid hormone in follicular fluid are related to the development of persistent follicles, this study is designed to evaluate the effect of administered adrenocorticotrophic hormone (ACTH) to weaned pigs on their ovarian steroidogenesis capacity and LHR expression.

**Methods:**

Ten multiparous sows were weaned and randomly divided into two groups (*n* = 5 each). Sows received 1 IU/kg ACTH (ACTH group) or saline (control group) every 8 h from days 3–9 after jugular vein intubation. Blood samples were collected throughout the experiment, and ovaries were collected after slaughter on day 10. Follicular fluid (FF) was used to determine the steroid hormone concentrations. The ovarian follicle wall was obtained and stored in liquid nitrogen to detect mRNA levels.

**Results:**

The plasma cortisol concentration was significantly (*P* < 0.01) elevated after ACTH injection. The estradiol (E_2_) and androstenedione (ASD) concentrations in FF were significantly lower (*P* < 0.05) in the ACTH group than in the control group. The LHR, 3β-hydroxysteroid dehydrogenase (3β-HSD), cytochrome P450 aromatase (P450arom), and cytochrome P450 17a-hydroxylase (P450c17) mRNA levels were significantly (*P* < 0.05) reduced in the ACTH group. The steroidogenic acute regulatory protein (StAR) level and cytochrome P450 side-chain cleavage (P450scc) was lower in the ACTH group than in the control group, but the difference was not statistically significant (*P* > 0.05). Immunostaining results revealed 3β-HSD,P450c17, and LHR expression in theca cells, and P450arom expression in granulosa cells. Immunohistochemical staining showed significant differences in the distribution of 3β-HSD, P450c17, LHR, and P450arom between the two groups.

**Conclusions:**

These findings indicated that ACTH significantly diminished the LHR expression and steroidogenesis capacity of the ovaries of weaned sows.

## Background

Stress is a well-known factor influencing reproductive performance in animals [1] Stressors come in various forms, and events such as weaning, grouping, and transportation have all been shown to influence reproduction in pigs and the pigs’ response to each of these stressors is different [2]. Responses to stress vary in response to factors such as type of stressor, duration, intensity, and individual variation between pigs [1–3]. It is difficult to compare and evaluate the different stressors that affect pig reproduction, particularly in long-term situations. A previous study found that ovarian cysts are an important disorder for sows, which accounted for 10% of reproductive problems in pigs [4]. However, little is known about ovarian cysts, because it may happen without the performance of reproduction confusion [4]. Although it is difficult to diagnose ovarian cysts, it is established that endocrine disruption is associated with ovarian cysts [5]. The confusion of endocrinological mechanisms plays a role in infertility by altering the physiology of gonadal tissues [5]. Stress results in alterations in hormonal events along with the immediate endocrine; some studies have reported that endocrine imbalance is the primary reason for the occurrence of ovarian cysts [6, 7]. Although stress-inducing practices such as sow crating and tethering have been gradually replaced by group penning to improve animal welfare, group penning can also give rise to stress among pigs due to animals pushing, riding, and biting one another [8]. Thus, stress is inevitable and difficult to evaluate. In pig production, many events can potentially cause stress and it is difficult to assess the precise relationship between stress and reproduction due to the varied number and type of stressors.

A series of studies have shown that reproduction in pigs is always impaired by stress through the neuroendocrine system [1, 9–11]. Without exception, various types of stress can activate the hypothalamic-pituitary-adrenal (HPA) axis, affecting hormone release and ovarian function [12]. Activation of the HPA axis suppresses gonadotropin secretion and influences ovarian activity by increasing the production of glucocorticoids [12]. The HPA axis is activated in response to stress, triggering the release of corticotrophin-releasing hormone (CRH) and vasopressin by the hypothalamus. Increased levels of CRH lead to increased ACTH release from the anterior lobe of the pituitary [2, 5]. ACTH acts on the adrenal cortex, stimulating the release of glucocorticoid hormones (e.g., cortisol in pigs) and influencing the physiological state of the sexual organs and steroid hormone pathways [13]. Exogenous ACTH administration has been shown to affect not only the adrenal glands but also the reproductive organs and several hormonal pathways either directly or by alterations in the cortisol level [14]. At ovulation, increased estradiol-17β (E_2_) and decreased progesterone (P_4_) concentrations in the plasma lead to increase in the frequency of GnRH secretion, contributing to the preovulatory LH surge [10]. Steroid hormones play a role in ovarian structures remote from the luteal or follicular cells where they are produced, and the process is realized by endocrine mechanisms [5, 15]. Decrease in the levels of steroid hormone can influence follicular development and even ovulation [16–18]. In general, androgens are secreted in granulosa cells and converted to E_2_ by P450arom; androgen levels are higher when the expression of the aromatase is suppressed, and this contributes to the formation of ovarian cysts [19]. Increased plasma cortisol levels lead to a decrease in the expression of steroidogenic enzymes and impair the synthesis capacity of E_2_ [20]. When the plasma E_2_ concentration is reduced, the positive feedback effect on GnRH/LH secretion is weakened and the preovulatory LH surge is then suppressed, ultimately affecting the success of ovulation [21].

ACTH administration did not significantly alter the plasma E_2_ and LH concentrations compared to the control group [13]. A review reported that no significant differences were found in the LH levels between control animals and experimental animals injected with ACTH, but the interval of the LH peak to ovulation showed a significant difference. The time from LH peak to ovulation was longer in the ACTH group than in the control group [12]. LH functions by binding to its receptors, and the LHR is the primary medium by which LH affects follicle development and maturation by stimulating the synthesis and secretion of steroid hormones [22].

Therefore, we aimed to study the effect of ACTH injection on the development and function of ovarian follicles; androstenedione levels; progesterone and E_2_ levels in follicular fluid (FF); LH receptor expression; and the expression of steroidogenic enzymes such as P450scc, P450c17, P450arom, StAR, and 3β-hydroxysteroid dehydrogenase (3β-HSD) in granulosa and theca cells of the ovarian follicle. To this end, we induced long-term stress in weaned sows by ACTH administration for 7 days.

## Methods

### Animals

Ten multiparous Suhuai sows at parity 3–4 and weaning at 28 d were selected for the experiment. The average weight of the tested sows was 175 ± 25 kg. The animals were housed in individual pens, fed twice a day, and provided with water *ad libitum*.

## Experimental design

### Grouping and jugular vein catheter

The jugular vein was cannulated on the weaning day and defined as day 1 of the experiment. Sows were fixed to a test bench after they were sedated with thiopentone sodium. A cannula was passed into the exposed jugular vein, while the free end of the cannula was passed subcutaneously and fixed behind the ear with adhesive bandage. After the surgery, the sows were randomly divided into two groups: an ACTH group and a control group (*n* = 5 per group). From days 2–8 after the surgery, animals in the ACTH group received 1 IU ACTH/kg body weight every 8 h. The ACTH was obtained from porcine pituitary glands (A6303, Sigma, USA). Sows in the control group received the same dosage of saline (0.09%) on the same schedule as the ACTH group. The cannula was filled with heparinized saline (0.09%) to prevent coagulation.

### Blood sampling

Blood samples were collected every 3 h starting from 9:00 am on day 2 until pro-estrus. After the start of estrus, blood samples were then collected every 1 h. The blood samples (10 mL) were collected into a heparinized vacuum blood tubes, centrifuged for 10 min at 1000 *g*, and stored at −20 °C until analysis.

### Collections of FF and follicle tissues

Porcine ovaries were collected within 10 min of slaughter of weaned sows. Follicles were then stripped from the ovary using sterile scissors and placed on ice. FF without blood was collected from each single follicle, and then stored at −80 °C for P_4_, E_2_, and androstendione (ASD) assays. Meanwhile, follicular tissues were stripped from the follicles using sterile scissors, and the tissues were then stored at −80 °C for extraction and analysis of mRNA.

### Detection of cortisol, P_4_, E_2_, and ASD levels in FF

The P4, E_2_, and ASD levels in follicular fluid were determined by ELISA assay kits (H089 and H102, Jiancheng, Nanjing, China; and 33–33720, TIANDZ, Beijing, China respectively) and the plasma cortisol level was evaluated by the same method (ANG-E31032P, Aoqing, Nanjing, China). All FF and plasma samples were centrifuged at 3000 rpm for 20 min at 4 °C. After centrifugation, all samples were diluted 1:5 in sample diluents and detected at 450 nm using a multimode reader (Infinite 200 PRO; Tecan, Geneva, Switzerland). The E_2_:P_4_ ratio was calculated after the E_2_ and P_4_ levels were detected in follicular fluid.

### Fluorescence quantitative real-time PCR

Total RNA was isolated using TRIzol reagent (Trizol-RNAiso Plus reagent, D9108A, Takara, China), and reverse transcription was carried out using Transcript Moloney murine leukemia virus kit (Invitrogen, Shanghai, China). For total RNA collection, 100 mg of follicle tissue was homogenized with 1 mL RNA extraction buffer (TRIzol; Takara Biotechnology, co., Ltd.) in a Super Fine Homogenizer (623003, Fluko, Germany). Once the total RNA was extracted, its concentration was determined by measuring absorbance in a spectrophotometer (M200PRO; Tecan, Austria) at 260 nm. The sequences of the *lhr, star, p450scc, p450arom, p450c17, 3β-hsd* were obtained from the National Center for Biotechnology Information Genbank. Table 1 presents the sequences and sizes of the *lhr, star, p450scc, p450arom, p450c17,* and *3β-hsd* genes.

**Table 1:** Sequences and sizes of all tested genes

Gene	Reference sequence	Primer sequence (5’-3’)	Size
*LHR*	NM-214449.1	sense GCTCACCCAAGACACTCC	190
		antisense CACATGAGGAAACGAGGC	
*StAR*	NM-213755.2	sense GGCAAGGCTCTTCTAACT	99
		antisense TAGACACGAAAGGGCTCA	
*P450scc*	NM-214427.11	sense CCAGCATTACCAGAAGCC	92
		antisense GAGCCATTACCTCCGTGT	
*P450arom*	NM-214429.1	sense AAGAAGGGTCACAACAAG	165
		antisense AAGAAAGCCAGTGAGCAG	
*P450c17*	NM-214428.1	sense ATGATCCAAGCCAAGACG	140
		antisense TTTACCACAGAGGCAGAAG	
*3β-HSD*	NM-001004049.1	sense CCTGGCAAGTATTTCTCGG	107
		antisense CCAGCAACAAGTGGACGAT	
*β-actin*	NM-001167795.1	sense CTCCATCATGAAGTGCGACGT	114
		antisense GTGATCTCCTTCTGCATCCTGTC	

### Immunohistochemical staining

Ovaries of the tested sows were obtained within 10 min of slaughter. They were then fixed in 4% formalin solution buffer (pH 7.4) for 24 h, and embedded in paraffin. Serial 5-μm-thick sections of ovarian follicles were cut and the sections were mounted onto 3-aminopropyltriethoxysilane (APES)-coated slides.

Sections were deparaffinized in xylene and dehydrated in an ascending ethanol series, and washed three times in PBS for 3 min each time. Antigen retrieval was carried out in 0.01 M sodium citrate buffer (pH 6.0) in a microwave oven, and the sections were rinsed three times with PBS for 3 min each time. The sections were then immersed in 3% hydrogen peroxide for 10 min to inactivate the endogenous peroxidase at room temperature. After inactivation, antigen retrieval was carried out in a microwave oven and nonspecific binding was blocked with 5% BSA. The following primary antibodies were used: anti-CYP19A1 (1:100, A1336, ABclonal Technology, Wuhan, China), anti-CYP17A1 (1:100, A1373, ABclonal Technology, Wuhan, China), anti-3βHSD (1:100, sc-30820, Santa Cruz Biotechnology, CA, USA), and anti-LHR (1:100, K-15, Santa Cruz Biotechnology, CA, USA). For the negative controls, 1% bovine serum albumin was used instead of the primary antibody. Next, sections were incubated with horseradish peroxidase (HRP)-conjugated goat anti-rabbit lgG (H + L; 1:150 dilution, IH-0011; Dingguo, Beijing, China) for 1 h at 37 °C. The reactions were visualized by treatment with diaminobenzidine (DAB) (AR1022, Boster, Wuhan, China) for 15 s.

Image-Pro Plus 6.0 software was used to evaluate the staining intensity by the average optical density (AOD). Ten typical fields were selected to estimate the percentage of immunopositive cells, and each field was examined twice by three authors.

### Statistical analysis

Data of the ACTH and control groups were analyzed by independent sample *t* test using Statistical Package for Social Science software (SPSS version 20.0 for Windows). All data are represented as the mean ± standard deviation (SD) and a P value of <0.05 was considered statistically significant.

## Results

### Plasma cortisol concentrations in the ACTH and control groups

**Fig. 1:**
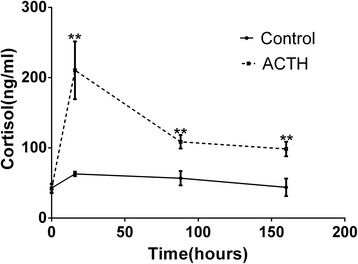
Plasma cortisol concentrations at four time points in sows of the control and ACTH groups. Plasma cortisol concentrations at four different time point (0, 16, 88, and 160 h) in the ACTH and control groups. After ACTH injection, the cortisol concentrations showed a significant increase (*P* < 0.01). **Values were statistically different at *p* < 0.01 compared to the control group

We evaluated the plasma cortisol levels in sows in the ACTH and control groups at four different time points in order to evaluate their stress levels (Fig. 1). The plasma cortisol levels were significantly elevated (*P* < 0.01) in the ACTH group than in the control group at 16, 88, and 160 h after ACTH injection. The cortisol level in the ACTH group was the highest at the second tested time point (16 h) compared to the other time points in the ACTH group (0, 88, and 160 h), and this difference was significant (*P* < 0.01). The cortisol concentration in the ACTH group declined sharply at 88 h. Thereafter, the cortisol concentration remained stable (from 88–160 h) and remained higher than the corresponding levels in the control group. The results revealed that plasma cortisol levels were maintained at a high level in the ACTH group, indicating successful stress induction in the ACTH model group.

### Steroid hormone levels in follicular fluid

**Fig. 2:**
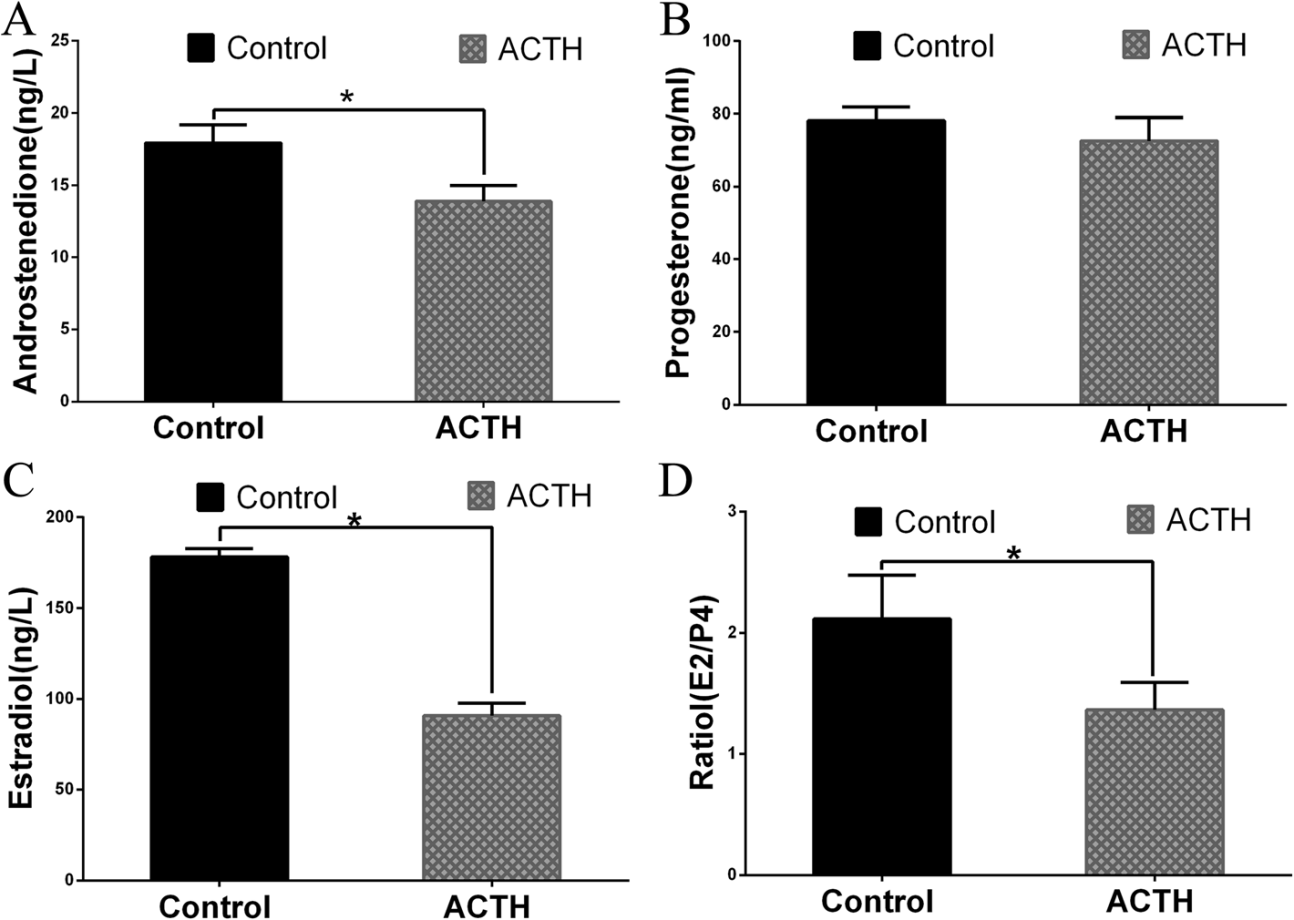
Changes in steroid hormone concentrations in ovarian follicular fluid Changes in the concentrations of (**a**) androstenedione (SD), (**b**) progesterone (P_4_), (**c**) estradiol (E_2)_, and (**d**) the E_2_/P_4_ ratio in the follicular fluid of weaned sows with and without ACTH. After ACTH injection, the ASD, E_2_, and E_2_/P_4_ ratio were all significantly decreased (*P* < 0.05) compared to the corresponding values in the control group. *Values were statistically significant at *p* < 0.05 compared to the control group

Figure 2 shows the concentrations of steroid hormones such as E_2_, ASD, and P_4_ in the ovarian follicular fluid of sows treated with ACTH for 7 days and control sows. The results revealed that the E_2_ and ASD levels had significantly decreased in the ovarian follicular fluid of the ACTH group (90.6 ng/L and 14.8 nmol/L, respectively; *P* < 0.05) compared to the corresponding levels in the control group (177.8 ng/L and 17.9 nmol/L, respectively). The P_4_ concentration of the ACTH group did not significantly decrease from that of the control group (71.5 vs. 80.2 ng/mL, respectively; *P* > 0.05). Although the E_2_/P_4_ ratios in both groups were >1, it was lower in the ACTH group than in the control group (Fig. 2d).

### Relative mRNA transcription levels of steroidogenic enzymes and LHR

**Fig. 3:**
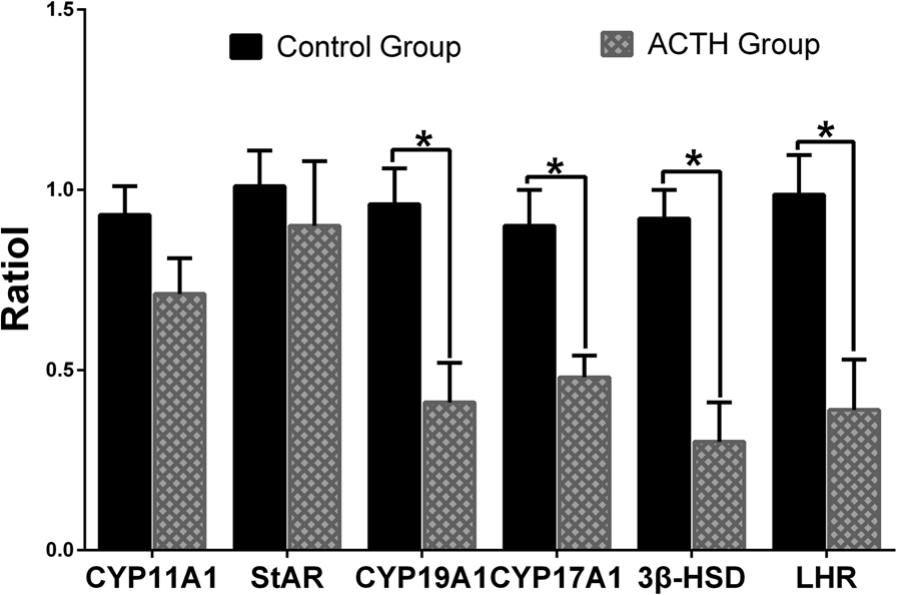
mRNA levels of steroidogenic enzymes and LHR Effect of ACTH treatment on the transcription levels of *star, p450scc, p450arom, p450c17, 3β-hsd,* and *lhr* relative to the *β-actin* transcription level in the follicular wall of the control and ACTH groups. *Differences in values were statistically significant at *p* < 0.05 compared to the control group

We then analyzed the corresponding mRNA transcription levels such as *cyp11a1*, *cyp17a1*, *cyp19a1, 3β-hsd, star, and lhr* by real-time quantitative polymerase chain reaction (RT-PCR). The transcription levels were normalized to the corresponding *β-actin* levels (Fig. 3). All the tested genes were associated with steroidogenic enzymes showed a lower transcription levels. The results revealed that the transcription levels of *lhr, 3β-hsd, cyp17a1,* and *cyp19a1* were significantly decreased (*P* < 0.05) in the ACTH group. The *cyp11a1* and *star* mRNA levels showed a similar trend; however, there was no significant reduction in their levels between the two groups (*P* > 0.05).

### Immunohistochemical staining

**Fig. 4:**
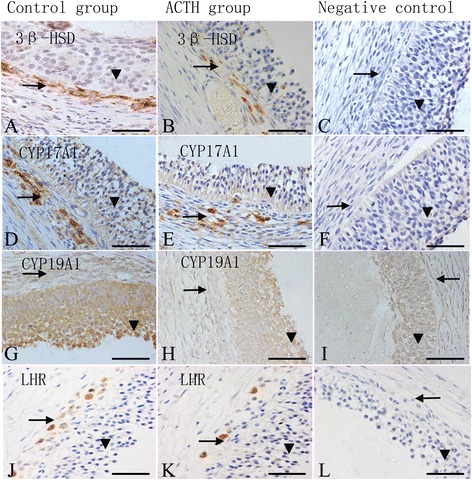
Localization of 3β-HSD, CYP17A1, CYP19A1, and LHR in sow ovaries Immunohistochemical staining of sow ovaries. Samples are counterstained with Mayer’s hematoxylin. Bars = 50 μm. The immunolocalization of 3β-HSD (**a**, **b**), CYP17A1 (**d**, **e**), CYP19A1 (G, H), and LHR (**j**, **k**) in the follicular walls of sows during the control and ACTH groups. The column on the right illustrates the immunohistochemical staining of the respective negative controls: (**c**) 3β-HSD, (**f**) CYP17A1, (**i**) CYP19A1, and (**l**) LHR. The expression levels of 3β-HSD (**a**, **b**), CYP17A1 (**d**, **e**), and LHR (**j**, **k**) were intense in theca cell layers (→) of both the control and ACTH-treated sows. Note the intense reactivity for CYP19A1 (**g**, **h**) in the granular cell layers (▼) of both the groups. ACTH group: After 7 days of ACTH treatment, the expression level of 3β-HSD (B), CYP17A1 (**e**), LHR (**k**), and CYP19A1 (**h**) were significantly reduced compared to the control group

Figure 4 shows the distributions of CYP17A1, CYP19A1, and 3β-HSD in the follicular wall of sow ovaries in both the groups. Immunohistochemical staining revealed that 3β-HSD and CYP17A1 are primarily localized in the theca cells. Meanwhile, positive staining of CYP19A1 is detected in granulosa cells, while some weak signals are observed in theca cells. Positive staining for LHR is observed in the ovarian theca cells in both the groups.

### Immuno-expression level

**Fig. 5:**
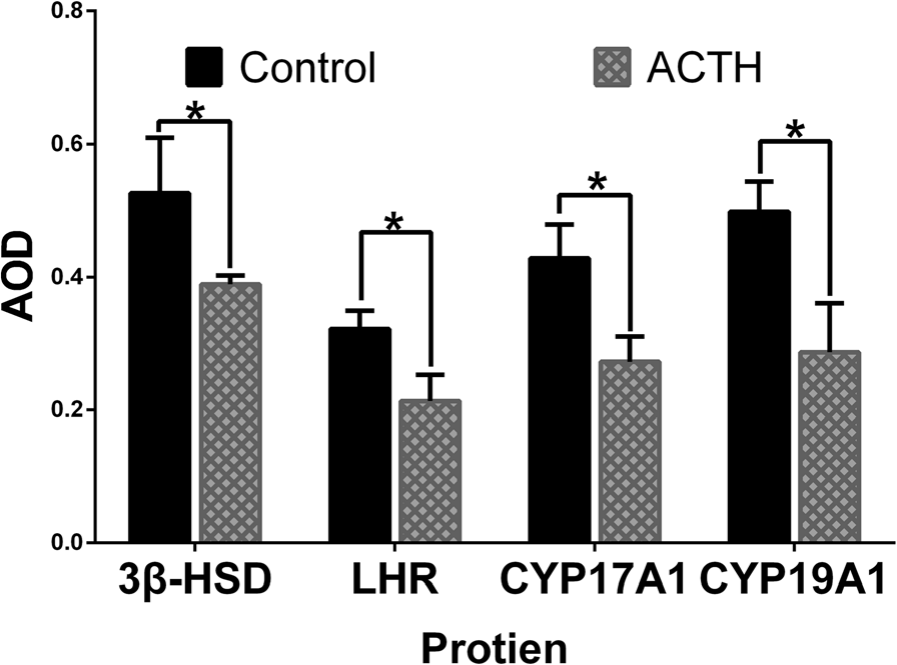
Changes in the average optical density of 3β-HSD, CYP19A1, CYP17A1, and LHR in the follicular wall After ACTH treatment for 7 days, weaker CYP17A1, CYP19A1, 3β-HSD, and LHR signals were detected in the follicular wall, and these signals were significantly decreased in the ACTH group than in the control group (*P* < 0.05). *Differences in values were statistically significant at a p value of <0.05 compared to the control group

Based on the AOD, the immunoreactivities of 3β-HSD, CYP19A1, CYP17A1, and LHR are significantly reduced in the theca cells of the ACTH group compared to those of the control group (P < 0.05; Fig. 5). The AODs of 3β-HSD and CYP19A1 are higher than those of CYP17A1 and LHR.

## Discussion

Although it is commonly known that stress induces disorders in follicular development and ovulation, the underlying mechanism remains unclear. ACTH administration has been reported to simulate the stress response, which is reflected by the plasma cortisol response, and influenced reproduction in pigs [8, 9, 11, 13, 23, 24]. In the present study, we administered sows with repeated ACTH injections to mimic stress; this resulted in an increase in the plasma cortisol concentration in the sows that were treated with ACTH but not in the untreated control sows, indicating successful establishment of a stress model. Previous studies mainly focus on the changes in the hormone levels in plasma, but hormone levels were not obviously affected by ACTH administration [8, 13]. Therefore, in our study we investigated changes in the steroid hormone levels in follicular fluid and LHR expression in the ovarian follicular wall in the stress model.

Cortisol is a sensitive indicator of stress, and our study found that sows with ACTH-induced stress had much higher plasma cortisol concentrations than control sows; our findings are in agreement with those reported previously [2, 25]. In addition to the higher plasma cortisol concentration, sows in the ACTH group also showed reduced E_2_ and ASD levels in FF and lower E_2_/P_4_ ratio compared to the corresponding values in the control group. Interestingly, the P_4_ concentration in FF did not significantly differ between the two groups. Ovarian steroidogenesis is known to be a multistep process involving many key enzymes [26]. The above results indicate that ACTH may not significantly affect the transcription of certain key enzymes. Cortisol, which is released by adrenal glands due to exogenous ACTH, is capable of damaging ovarian function [2, 27, 28]. ACTH, which is secreted from the pituitary gland, can induce cortisol secretion by acting on the adrenal gland [10, 25]. Increase in the plasma cortisol concentration would initiate the negative feedback of E_2_, which would then suppress the frequency or amplitude of GnRH, reducing the secretion of gonadotrophin [28]. At the hypothalamo-pituitary level, gonadotrophin inhibition influences ovulation and triggers the formation of small ovarian cysts [24, 29]. Hormonal interaction of the HPA and hypothalamic-pituitary-gonadal (HPG) axes contributes to reproductive problems in female pigs [5]. Increased cortisol concentrations can suppress the synthesis of steroid hormones and can even impair the preovulatory LH surge, resulting in ovulation failure [27, 28]. Suppression of the LH surge can impair follicle development and estrogen synthesis as well as increase the formation of follicular atresia [17]. Steroid hormone release is controlled by endocrine mechanisms, and changes in the concentrations of steroid hormones will influence reproductive function [5]. Steroid hormones are important factors affecting ovarian follicle growth and ovulation in weaning sows. As paracrine/autocrine agents, these hormones can play roles on or within the cells where they were produced [15].

Ovarian steroidogenesis is a multistep process involving several key enzymes [26]. Enhanced E_2_ biosynthesis ability is always accompanied by upregulation of LHR and some steroidogenic genes [30]. In the present study, the follicular fluid of the ACTH group had reduced E_2_ and ASD concentrations. Accordingly, the *lhr*, *3β-hsd, cyp19a1,* and *cyp17a1* mRNA levels were also reduced in the ACTH group compared to the control group; this reduction may be associated with the high plasma cortisol levels induced by ACTH. The frequency or amplitude of GnRH could be suppressed with the increased in the plasma cortisol level [28]. In addition, treatment with a GnRH receptor antagonist has been shown to decrease LH secretion and reduce the *lhr*, *cyp19a1*, and *cyp17a1* mRNA levels [31]. Compared to the control group, the ACTH group had a lower E_2_/P_4_ ratio, and the *lhr*, *cyp19a1*, and *cyp17a1* levels were lower in estrogen-inactive follicles than in estrogen-active follicles [32]. The mRNA and protein levels of these enzymes were related to the biosynthesis and transfer of androgens. In previous literature, 3β-HSD was shown to convert pregnenolone into P_4_ and transfer this hormone outside the mitochondria, while P450c17 was shown to produce ASD via 17-hydroxylation of P_4_ [33]. The results of the immunohistochemical staining in our study revealed positive signals of P450c17 and 3β-HSD distributed in theca cells, and these positive signals were reduced in the ACTH group compared to the control group. When the 3β-HSD expression level is reduced, it interferes with the ability of P_4_ transfer, resulting in ASD biosynthesis and reduced P_4_ concentration. Similarly, the suppression of P450c17 reduces the 17-hydroxylation activity of this enzyme, consequently affecting ASD biosynthesis [18]. Except for aromatization, which occurs in granulosa cells, all other processes of steroidogenesis occur in theca cells. P450arom is expressed in granulosa cells and is the last key enzyme that plays a role in the formation of estrogens from androgens [34]. Disruption of ovarian steroid synthesis in sows may be associated with defects in the aromatase complex, which influences hormone conversion in peripheral fat tissue [10]. Suppression of P450arom activity can reduce the E_2_ concentration in FF [35, 36]. In a study on women with polycystic ovary syndrome (PCOS), the P450arom expression levels in granulosa cells were very low and undetectable, indicating that the process of conversion of androgens into estrogens was disturbed, affecting the development of the ovarian follicle [37].

Estrogens can regulate the development of ovarian follicles by stimulating the proliferation of granulosa cells [38]. Decrease in the estrogen levels could influence the LH surge [39] and the LHR expression in the ovary [22]. LHR expression is a vital mediator for the various functions of LH in sows, in including ovulation. LH can influence the P_4_ and ASD production and induce ovulation by binding to the LH receptor [40]. A previous study reported that inactivation of LHR by gene mutation affected follicular development at all stages [41]. Glucocorticoids have been shown to influence the responsiveness of gonadal to LH and the expressions of LHR [42]. LHR production increases with follicle development and matures before ovulation [43] while the expression would be influenced by many elements such as the circulating LH levels [31]. The cortisol that is produced in response to stress suppresses GnRH secretion and decreases the LH level [28]. The ACTH-induced increase in the plasma cortisol level affects the LHR expression in ovarian follicular walls. In addition, LHR expression is related to estrogen activity, and LHR expression has been found to be lower in estrogen-inactive follicles than in estrogen-active follicles [32].

## Conclusions

The present study demonstrated that higher cortisol levels, induced in response to stress, affected the growth and ovulation of follicles. ACTH injection over long-term increased the plasma cortisol concentration, which reduced the steroidogenic hormone levels and LHR expression in the ovaries of weaned sows.

## Abbreviations

3β-HSD: 3β-hydroxysteroid dehydrogenase
ACTH: Adrenocorticotrophic hormone
AOD: Average optical density
ASD: Androstenedione
BSA: Bovine serum albumin
CRH: Corticotropin releasing hormone
CYP11A1: Cytochrome P450 family 11 subfamily A member 1
CYP17A1: Cytochrome P450 family 17 subfamily A member 1
CYP19A1: Cytochrome P450 family 19 subfamily A member 1
E2: Estradiol
FF: Follicular fluid
GnRH: Gonadotropinreleasing hormone
HPA: Hypothalamic-pituitary-adrenal
HPG: Hypothalamic-pituitary-gonadal
LH: Luteinizing hormone
LHR: Luteinizing hormone receptor
mRNA: Messenger ribose nucleic acid
P4: Progesterone
P450arom: Cytochrome P450 aromatase
P450c17: Cytochrome P450 17a-hydroxylase
P450scc: Cytochrome P450 side-chain cleavage
PBS: Phosphate buffer saline
StAR: Steroidogenic acute regulatory protein.
